# Influence of Sociodemographic Factors and Incontinence Care Practices on the Development of Incontinence-Associated Dermatitis

**DOI:** 10.3390/jcm15051752

**Published:** 2026-02-25

**Authors:** Maria Piedad Garcia-Ruiz, Rosa Maria Torres-Bautista, Maria Dolores Lopez-Franco, Agustina Orozco-Cuadrado, Vicenta Nava-Anguis, Araceli Alarcon-Juarez, Francisco Pedro García-Fernández

**Affiliations:** 1 Jaén Norte Health Management Area, 23700 Jaén, Spain; 2 UGC Linares C “San José”, 23700 Jaén, Spain; 3 Department of Nursing, Faculty of Health Sciences, University of Jaén, 23071 Jaén, Spain

**Keywords:** incontinence-associated dermatitis, non-irritating barrier films, zinc oxide, effectiveness, prevention, incontinence, skin protection, hygiene, cleanliness, absorbents, moisture-associated skin damage

## Abstract

**Objectives:** The general objective of the study was to analyze the influence of sociodemographic factors and care and hygiene practices on the appearance of Incontinence-Associated Dermatitis (IAD). The specific objectives were to identify the relationship between sociodemographic variables (age, sex, comorbidities) and the incidence of IAD, and to evaluate hygiene protocols (cleaning products and absorbent use and practices) and compare time to IAD onset according to the hygiene practices used. **Methods:** A multicenter, prospective cohort study was conducted across 10 social health centers. The study population consisted of older institutionalized patients with urinary and/or mixed incontinence who used absorbents continuously. The variables included risk assessment using validated scales, such as the Braden scale and the Perianal Assessment Tool (PAT), as well as ad hoc questionnaires. Survival analysis of time-to-event onset was assessed using the Kaplan–Meier curve and the Log-Rank test. **Results**: A statistically significant association was found between the occurrence of IAD and the Braden scale (*p* = 0.004) and the PAT scale (*p* = 0.02). However, no statistically significant association was found with age, sex, or the Barthel Index. Regarding hygiene, soapy wipes were associated with the highest incidence of IAD. In contrast, dry wipes were the most effective, with the lowest cumulative risk and the longest time-to-event (*p* = 0.001). The brand of the absorbent used had a significant influence (*p* = 0.024), suggesting that the “B” brand prevented one IAD per six patients compared with the “A” brand. The frequency of absorbent changes did not show a statistically significant association. **Conclusions:** The risk of developing IAD was strongly linked to the scores of the Braden and PAT scales, the brand of the absorbent (“B” being more effective), and the cleaning product used, with dry wipes being the most protective compared to soapy wipes. On the other hand, factors such as age, sex, degree of dependence (Barthel), or frequency of absorbent change did not show a significant influence on the appearance of IAD.

## 1. Introduction

Incontinence-Associated Dermatitis (IAD) is the most common subcategory of Moisture-Associated Skin Damage (MASD). MASD is defined as inflammation and/or erosion of the skin caused by prolonged or excessive exposure to moisture, such as urine and feces. IAD is a very relevant issue in health care, as it affects people in a situation of dependency (permanent or transitory), seriously compromising their quality of life and comfort [1,2,3,4].

Etiologically, IAD is a complex and multifactorial condition. Constant exposure to body effluents leads to overhydration and maceration of the stratum corneum, the outermost layer of the skin, thereby increasing susceptibility to damage. The key pathophysiological mechanisms center on inflammation, altered skin pH, and physical deterioration of the skin. Specifically, the breakdown of urea into ammonia alkalizes the skin’s pH, disrupting the protective dermolipid barrier and facilitating the proliferation of pathogenic microorganisms. In addition, liquid stools are particularly irritating due to digestive enzymes (proteases and lipases) that, in an alkaline environment, break down essential lipids and proteins in the stratum corneum [2,5,6,7,8,9]. The epidemiology of IAD shows prevalence rates ranging from 4.3% to 42% and incidence rates ranging from 3.4% to 50%, depending on the population and environment studied.

Numerous sociodemographic and clinical factors act as adjuvants or predisposing factors, increasing an individual’s risk of developing IAD. Advanced age is a crucial factor, given that aging skin is more fragile and has a compromised barrier function, with the incidence of IAD being higher in older people. Other risk factors include chronic health problems (such as diabetes), malnutrition or hypoproteinemia, fever, impaired consciousness, and immobility [2,5,6,8,9,10,11].

The prevention and treatment of IAD focus on a structured skincare program based on Hygiene, Hydration, and Protection [9]. Regarding care and hygiene practices, gentle, careful cleansing is recommended using products with a pH close to the skin’s physiological pH (4.0–6.8), such as specific leave-on cleansers, since traditional alkaline soaps can damage the skin barrier. The use of absorbents is also a determining factor, as the use of occlusive or inappropriate devices can prevent skin drying, increase sweating, and raise the skin’s pH, thereby increasing the risk of damage [5,6,7,9,10,12,13,14,15,16,17,18,19,20,21].

The lack of high-quality evidence has resulted in the implementation of care that is often based on personal experience or opinion, rather than standardized, science-based protocols. It is crucial to conduct studies that standardize care, optimize care time, and improve treatment effectiveness by comparing commonly used products.

Therefore, the present study aims to address this need for evidence by analyzing the influence of sociodemographic factors and care practices on the occurrence of IAD, focusing on the protective effects of the inputs used.

## 2. Objectives

General objective:

‑ To analyze the influence of sociodemographic factors and incontinence care and hygiene practices on the appearance of IAD.

Specific objectives:

‑ To determine if there is a relationship between sociodemographic variables (age, sex, comorbidities) and the incidence of IAD in the population studied.
‑ To determine if there is a relationship between hygiene practices (type of cleaning products and absorbent use practices) and the incidence of IAD.
‑ To compare the time to onset of IAD according to the incontinence care practices used, identifying in which group the event occurs earlier.

## 3. Methodology

Type of study. This research is part of a larger research project in which a multicenter prospective cohort study was carried out across 10 social health centers in several primary care districts in the provinces of Jaen and Cadiz, focused on the care practices used as prevention (zinc oxide and non-irritating barrier films) for moisture injuries associated with incontinence. In a previous article, the profile of the institutionalized patients with incontinence (urinary and/or mixed) included in this research project was presented [11]. In the present study, its influence on the development of IAD is investigated.

Study overview:

- Study population. Patients treated in nursing homes in the Jaen-South Jaen, North Jaen and Cadiz health districts who have urinary and/or mixed incontinence. These nursing homes care for dependent older adults, are accredited by the Autonomous Community (ORDER of 5 November 2007 regulating the procedure and requirements for the accreditation of centers for older adults in a situation of dependency in Andalusia), and have their own doctor and 24-hour nursing care.
- Sample: Patients from the study population who met the inclusion/exclusion criteria, who agreed to participate in the study, and who remained admitted from the date of the start of the study in the participating centers.
- Sample selection method: A non-probabilistic, intentional, convenience-type sampling was carried out, selecting all participants in the primary project from the beginning of the study until the sample size was completed.
- Inclusion criteria:
  ○ Be over 18 years of age and have a life expectancy of more than 6 months from the start of the study.
  ○ Have urinary incontinence and/or mixed incontinence.
  ○ Use of absorbents on a continuous basis and as the only method for managing incontinence.
  ○ Previous absence of IAD.
  ○ Regularly use only one of the products under study: zinc oxide ointment or non-irritant barrier film.
- Exclusion criteria:
  ○ Patients with allergies to some of the study products.
  ○ Patients in a situation of terminal illness.
  ○ Presence of pressure lesions, multifactorial lesions, or combined lesions prior to the start of the study in the gluteal, sacral, genital, or perigenital area.
- Sample size calculation: For the sample calculation in the cohort study, the hypothesis was taken into account that people treated with Non-Irritating Barrier Products (NIBPs) have better prevention outcomes in incontinence-associated dermatitis (IAD) than those treated with ZnO ointments. Patients were included according to the estimates made in the cohort study of the main project, which assumed an α error of 0.05, a statistical power of 80%, and considering that the IAD associated with patients with incontinence was 36% [22], establishing a sample size of 110 patients (55 per cohort). 95% confidence intervals were used.

Variables analyzed: The following variables were analyzed: age, sex, associated comorbidities, pharmacological treatment, nutritional degree using a validated scale such as the Mini Nutritional Assessment (MNA), type of incontinence, existence of urinary tract infection or gastroenteritis at the time of the intervention, degree of personal autonomy (Barthel), type of absorbent used, number of daily changes in absorbent, Perineal Injury Measurement Scale (Perianal Assessment Tool, PAT), Risk Scale for Dependence-Related Injuries (Braden scale), hygiene care practices used, bad habits, type of skin protection used after hygiene and percentage of ZnO in the case of using ZnO ointment as a protection measure, presence and categorization of MASD, and presence and degree of erythema.

Methods and instruments in data collection: Data collection was carried out through an ad hoc questionnaire and validated scales for those variables that had this option: Barthel Index, Perianal Assessment Tool, Braden scale, Visual Erythema Scale, and Categorization of MASD proposed by the National Group for the Study and Counseling of Pressure Ulcers (GNEAUPP). The outcome assessors were unaware of the participants’ hygiene care practices and of the absorbent brand or change frequency. They also did not know the protection method used, whether zinc oxide ointment or NIBP. The study maintained a simple blind. To ensure consistency and minimize variability between evaluators, all participating researchers received standardized training before the start of the study. This training focused on the correct application of the Braden and PAT scales, as well as the standardized classification of injuries, requiring participants to present a certificate of completion of the training course in injury identification (SECLARED). The assessment was carried out with four measurements during a six-week follow-up.

Data analysis: A global descriptive analysis was carried out. The qualitative variables were described using frequencies and percentages, and the quantitative variables were reported as means and standard deviations for normally distributed variables and as medians and interquartile ranges for non-normally distributed variables. To analyze survival or time-to-event, the Kaplan–Meier curve and the Log-Rank test were used. Due to the limited number of events per center (sparse data), stratification or adjustment by center was not performed to avoid model instability.

Ethical aspects: The study was approved by the Coordinating Committee on Biomedical Research Ethics of Andalusia (CCEIBA). Verbal and written consent from patients and/or relatives was obtained, in accordance with the Declaration of Helsinki, and identification data were coded and treated confidentially, as provided by Organic Law 3/2018, of 5 December, on Data Protection and ARCO Rights (Access, Rectification, Cancelation and Opposition). This study is also funded by the FIBAO (Foundation for Biomedical Research of Eastern Andalusia, Alejandro Otero) under Project code AP-0312-2022.

## 4. Results

1. On whether sociodemographic variables influence the appearance of an IAD.

All patients met the inclusion criterion of incontinence; however, in 58.5% of cases, they also had fecal or mixed incontinence.

To determine these associations, in addition to age and sex, three scales were used: Barthel for dependence, PAT for the risk of injury due to moisture, and Braden for the risk of pressure injuries, all of which have been related to the appearance of an IAD.

**Figure 1:**
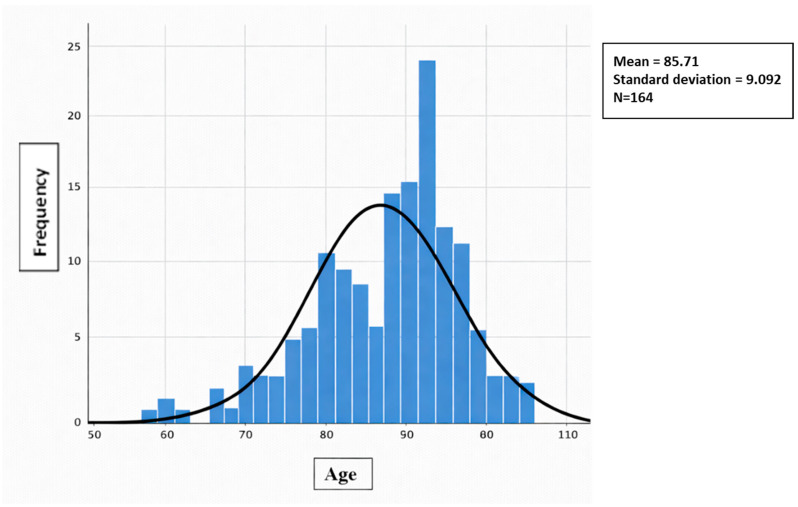
Distribution of the sociodemographic variable of age. Source: Authors.

**Figure 2:**
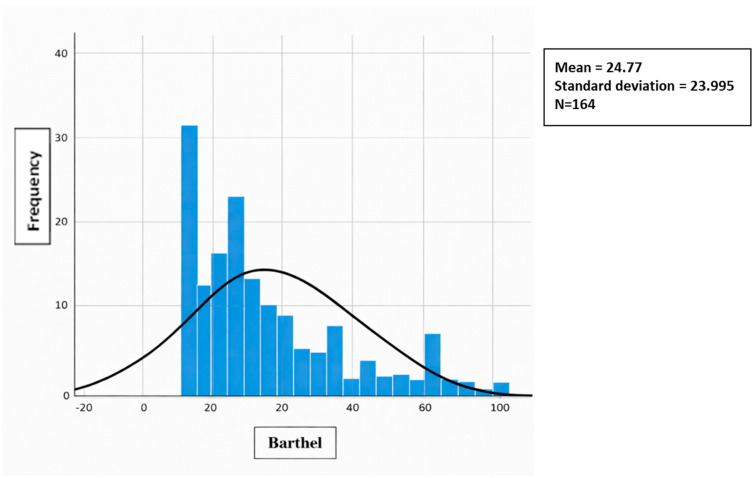
Distribution of the sociodemographic variable of the degree of dependency (Barthel Index). Source: Authors.

**Figure 3:**
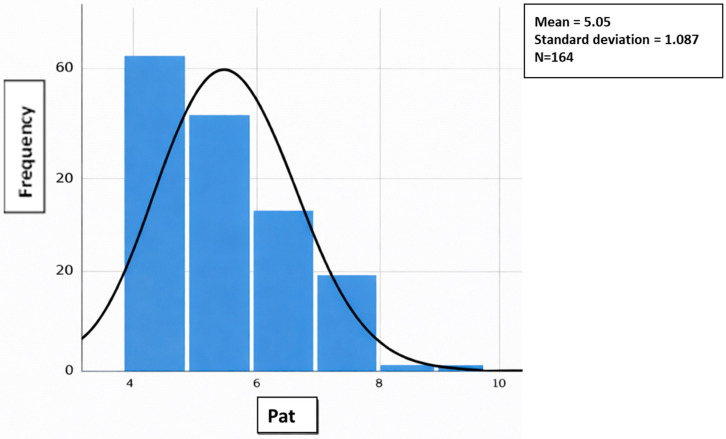
Distribution of the sociodemographic variable of risk of developing IAD (PAT). Source: Authors.

**Figure 4:**
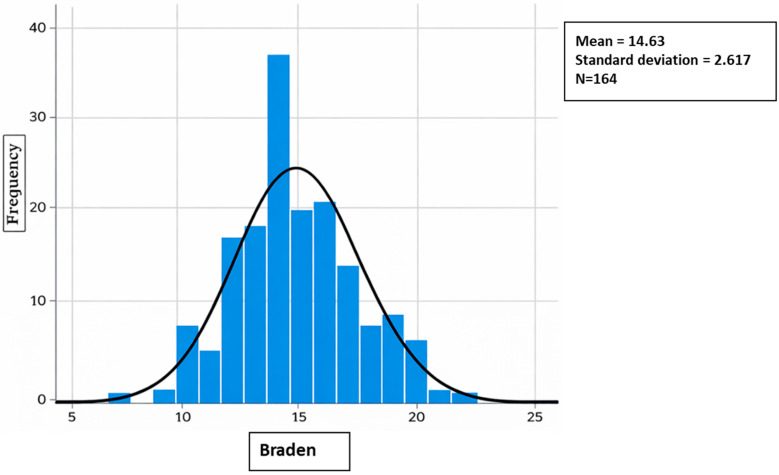
Distribution of the sociodemographic variable of pressure injury risk (Braden). Source: Authors.

All quantitative variables (age, Barthel, PAT, and Braden) did not have a normal distribution (Kolmogorov–Smirnov test with Lilliefors correction), so non-parametric means were used. This test was conducted using normal distribution charts. Figure 1, Figure 2, Figure 3 and Figure 4 show the distribution graphs of the sociodemographic variable of age, degree of dependence (Barthel Index), risk of developing IAD (PAT), and risk of pressure injuries (Braden), respectively. As shown in the graph, the curve is asymmetric, positive, and platykurtic.

Table 1 and Table 2 present the frequencies and mean values for each variable analyzed, distinguishing between the IAD and non-IAD groups, and report the statistical significance of each association after adjustment for confounding.

The association between the Braden scale and the PAT scale, and the occurrence of IAD, is statistically significant. The mean scores on the Braden scale for patients who developed lesions were 13.62 (SD = 0.373), and on the PAT scale, 5.34 (SD = 0.163).

Thus, the greater the risk detected in the appearance of pressure lesions (Braden scale) and the greater the risk of developing a skin lesion due to incontinence in the perineal area (PAT Scale), the greater the likelihood of IAD.

However, the study found no association between the Barthel Index and the development of an IAD, and it cannot be concluded that greater dependence is associated with a higher risk of developing IAD.

On the contrary, for the Barthel Index, the mean score of patients who developed a lesion was 4 points lower than that of those who did not; however, the high dispersion of the data with overlapping intervals indicates that this difference does not reach statistical significance.

2. On whether cleaning and moisture control products (absorbents and their frequency of changes) influence the appearance of an IAD.

When assessing hygiene, the following cleaning options were analyzed: dry wipes, 3-in-1 wipes and soap (specific for cleaning the genital area), standard soap and water, and soapy wipes. The brands and models did differ from one social health center to another, so this bias could not be avoided (as it was an observational study), but it was taken into account and measured independently.

A Chi-square test showed that soapy wipes had a statistically significantly greater association with the appearance of IAD (*p* = 0.001), with IAD developing in 57.6% of the patients where they used it. In other words, 1 in 1.7 patients develops IAD when their hygiene care practices involve soapy wipes.

The second-highest hygiene care practice in terms of IAD incidence was the use of dry wipes. In this group, 1 in 2 patients who used these developed IAD (50% of the total sample who used dry wipes).

**Figure 5:**
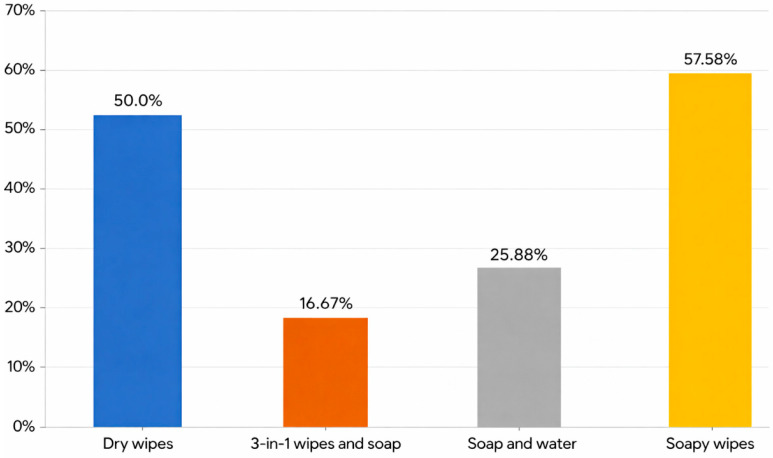
Hygiene care practice and the percentage of occurrence of IAD. Source: Authors.

The standard soap and water hygiene accounted for 25% of IAD incidents. The hygiene care practice found to be the most protective against IAD was 3-in-1 wipes and soap, products designed for genital hygiene, which do not require rinsing. 3-in-1 wipes and soap resulted in IAD in 16% of the care group; that is, only 1 person in 6 who used this hygiene method developed an IAD (Figure 5).

Regarding moisture control, this study considered only the continuous use of absorbents, without specifying a specific change frequency; however, this was accounted for and analyzed separately, as described below.

As it was an observational study, it was not possible to interfere with the brand and model of the absorbents; however, all the residences were contractually supplied with absorbents through the Andalusian Health Service, and the brand supplied was the same without possibility of change, with the exception of a single social health center that used another brand in all its patients. This center had a representative sample of the total and was able to be analyzed independently, as explained above.

The absorbent brand: “A” was present in 35.2% of patients who developed an IAD, and the absorbent brand: “B” was present in 16.7% of IAD cases. Analyzing this data with the Chi-square test yielded a statistically significant association (*p* = 0.024). This data corroborates when analyzed using Relative Risk, with a difference in risks between groups of 18.5% (95% CI 4.5–32.7%), or with the Newcombe test of differences in independent proportions, with 17.76% (CI 1.77–29.88), providing even more robust data. This leads us to the observation that, with a 95% CI of 4–23, for every 6 patients (NNT) who use the “B” brand, an IAD is avoided among those who use the “A” brand. The “A” brand corresponds to Incopack^®^ absorbents and the “B” brand to Lindor^®^ absorbents.

Finally, with respect to the frequency of changing absorbents and the appearance of an IAD, the statistical association, as measured by Student’s *t*-test, was not significant (*p* = 0.6). However, patients with IAD showed a higher mean frequency of change (3.94) than those without IAD, with a mean number of changes in absorbents of 3.62.

**Figure 6:**
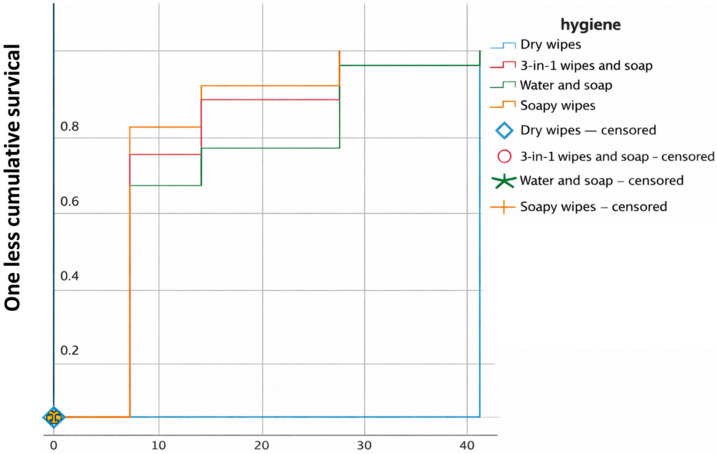
Kaplan–Meier curve for hygiene care practices. Source: Authors.

When performing an analysis using the Kaplan–Meier curve to assess the cumulative survival in relation to the time to onset in days or rather “One minus survival”, i.e., the cumulative probability of occurrence of an event (in this case the appearance of a lesion), with respect to the different hygiene care practices, we obtain the following results (Figure 6).

Dry wipes had the lowest event accumulation, indicating that, in this group, it took longer to develop; thus, they appear to be associated with a lower cumulative event rate (IAD) or a delay in their onset.

3-in-1 wipes and soap showed an accumulation of early events, suggesting that this option could be associated with a faster onset of the event. This care practice showed a greater accumulation of events over a shorter period, suggesting that it may be associated with an earlier onset of events, as with soap and water and soapy wipes.

Soap and water also showed an early accumulation of events, similar to 3-in-1 soap and dry wipes. However, soap and water were associated with adverse events throughout follow-up, unlike the other hygiene practices. Therefore, it can be argued that, if the incontinence is due to an acute process that will last a short time, the hygiene care practice could be indistinct. Still, if a long period of time is foreseen or involves a chronic process, the hygiene care practice is very influential, with clear differences in the long term.

These differences were statistically significant, as determined by the Log-Rank test (*p* = 0.001).

**Figure 7:**
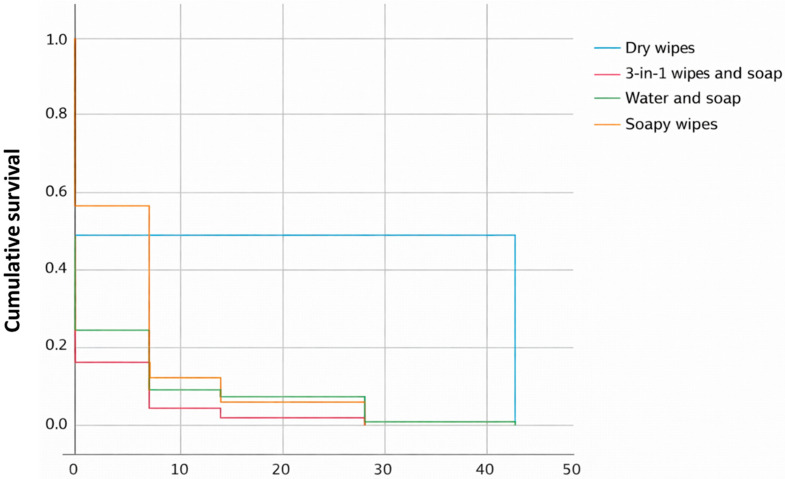
Kaplan–Meier curve for hygiene care practices, according to survival. Source: Authors.

Similarly, in Figure 7, which shows the Kaplan–Meier curve for cumulative survival, we observe that dry wipes have the longest time to the onset of the event (IAD).

The dry wipes group (blue) stands out clearly. Cumulative survival remains constant at 0.5 (50%) over an extended period, up to approximately 42 units of time, at which point it drops to 0.0 by study completion. The remaining methods (3-in-1 soap and wipes, soap and water, and soapy wipes) show a much earlier and faster onset of the event.

**Figure 8:**
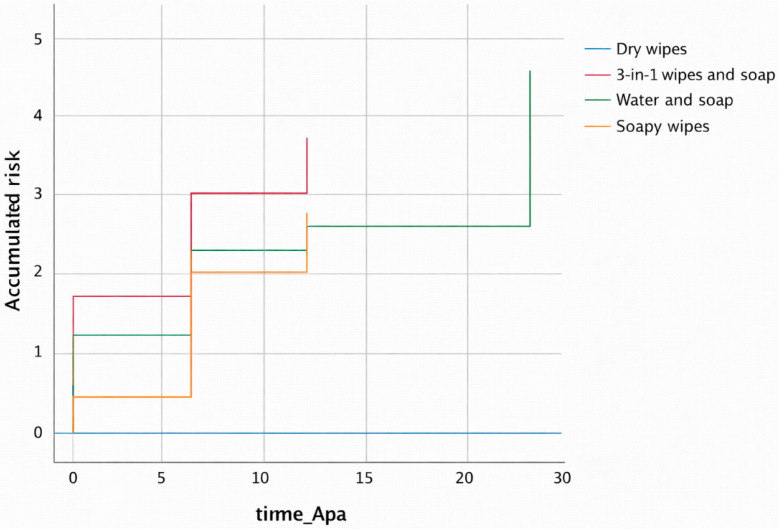
Kaplan–Meier curve for hygiene care practices, according to the risk function. Source: Authors.

In Figure 8, the direct counterpart of Figure 7, we see the Kaplan–Meier Curve analyzing its risk function.

This risk curve reinforces and complements the interpretation of the Kaplan–Meier survival curve. We observed that dry wipes clearly proved to be the most effective method, as they presented the lowest cumulative risk (zero) and the longest time to occurrence of the event. The methods of 3-in-1 wipes plus soap, soap and water, and soapy wipes had the highest risk during the study period, indicating an earlier occurrence of the event. The superiority of dry wipes suggests that this hygiene care practice best prevents or delays the onset of the event of interest in this study.

**Figure 9:**
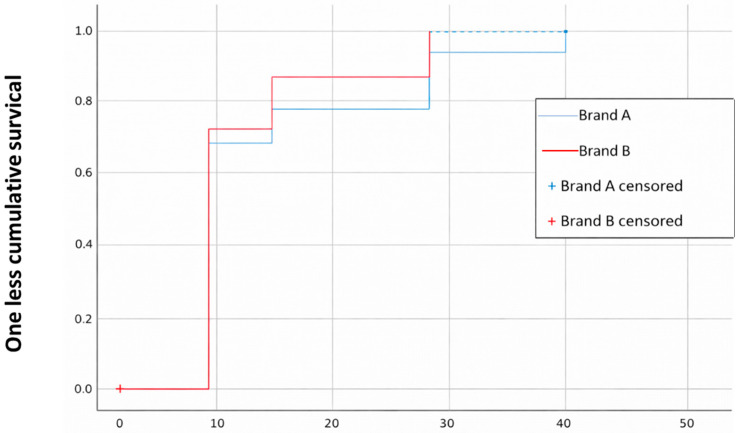
Kaplan–Meier curve for the absorbent brand. Source: Authors.

The absorbent brand was also analyzed using the Kaplan–Meier curve to assess the incidence over time (Figure 9).

The proportion of individuals who developed IAD and the time to onset are shown. The “B” brand showed a slightly earlier accumulation of events (IAD) than the “A” brand, suggesting that the event occurs earlier.

Although the difference between the two groups appears small, the Log-Rank test indicates a significant difference (*p* = 0.022).

Table 3 presents a summary of the sociodemographic variables and their statistical significance for ease of reference.

## 5. Discussion

Preventive measures used in patients with incontinence and their influence on the development of IAD.

Skin hygiene is a fundamental aspect in clinical nursing practice, with a substantial impact on the prevention and treatment of various skin conditions, especially MASD and IAD [23].

Traditionally, pH-neutral soap and water have been used for skin hygiene and care [24]. However, several studies and reviews suggest that skincare practices using traditional soap and water are less preferable or less effective for maintaining skin integrity and preventing IAD and xerosis cutis [23,25].

The frequent and repeated use of conventional soaps is considered too aggressive for skin exposed to moisture and for fragile skin [24]. This is because many common soaps are too alkaline (pH 9–10), which disrupts the skin’s acid mantle or damages its structural proteins [21]. When the skin becomes more alkaline, its ability to inhibit bacterial growth is compromised [12]. Traditional soap, combined with towel drying, can disrupt the skin’s barrier function. The soap and water formula often requires excessive rubbing or friction to remove urine and fecal residue, which can cause microdamage and abrasion of vulnerable skin [21,26,27,28]. In cases where the skin is already denuded, soap and water may not be the best option, as washing can further exacerbate the damage [29]. In addition, the surfactants present in soap can cause contact dermatitis, dehydrate the skin and damage keratinocytes since, although they promote cleaning, they can eliminate the lipid content of the stratum corneum, which weakens the skin’s protective function [26,30].

Although cleaning with soap and water may be effective in reducing microorganisms in the groin and perineum area [29], current evidence suggests that they represent too aggressive a technique for vulnerable skin exposed to moisture, and although soap and water are economic resources, a leave-in cleanser may be less expensive in terms of labor [23,24,31].

In contrast to soap and water, several reviews indicate that leave-in cleansers and wipes with moisturizing and protective properties are beneficial and often preferable [9,23,24,25,28,32]. Cleaning with this type of product should be performed using a gentle, careful technique with minimal friction, avoiding rubbing or scrubbing the skin [9,21,26,33]. The use of soft materials, such as disposable nonwoven towels, is recommended to minimize friction-related damage and prevent cross-infection [9,26,33]. These hygiene care practices can reduce nursing time and associated labor costs [9,24,34,35,36].

If we compare the incidence or prevalence data of our study with those of the scientific literature, we find similarities. A systematic review [31] found that a skincare protocol using wipes with 3-in-1 cleansing, moisturizing, and protective properties resulted in a lower incidence of IAD. Specifically, 80 cases of IAD per 1000 participants were reported among participants who used three-in-one wipes, compared with 259 cases per 1000 participants among those who used pH-neutral soap and water. This finding was based on one RCT with 121 participants, and we judged the evidence to be of moderate quality.

A 2022 review by Rumbo Prieto [24] aimed to establish the efficacy of topical skin products in reducing the development or severity of IAD, including the use of 3-in-1 wipes, NIBP, foam cleansers, leave-in cleansing lotion, and a long-lasting protective cream, and concluded with the need to replace traditional soap and water-based perineal care with protocols that incorporate specialized products that combine the cleansing, moisturizing and protecting the skin barrier [24].

Another review by Bohnenkamp showed that at 120 days, the prevalence of IAD in the group treated with 3-in-1 wipes was 8.1%, whereas in the soap and water group, it was 27.1% [32].

Dry wipes, with no rinsing afterwards, were the care practice in our study that showed the best results. As we have seen above, the Kaplan–Meier curve visually points to this hygiene care practice being superior (Figure 6, Figure 7, Figure 8 and Figure 9); however, it is essential to interpret this finding with caution due to the low number of events (only 4 cases) that defined the estimates of the Kaplan–Meier curve in the dry wipe hygiene group with a total of 164 participants in the study. While the direction of the result is clear, extrapolation of this potential benefit to a wider population should be the subject of more powered studies with a higher number of events. For the remaining hygiene care practices, the curve falls more quickly and lower, so the results are worse, indicating that most individuals developed IAD more quickly and more frequently. These results are in line with the literature, which positions this method as an advantage over more conventional ones, such as soap and water. They are defined as specialized cleansers with a slightly acidic pH (close to 5.5), designed to be gentle, do not require rinsing, and often contain surfactants and emollients or moisturizing ingredients [28,34,37]. A randomized controlled trial compared the use of a 3-in-1 disposable wipes (impregnated with a cleansing agent, a moisture barrier enhancer, and a dimethicone-based skin protectant) with standard care (cleaning with pH-neutral soap and water) in nursing home patients and participants who used the 3-in-1 wipes had a significantly lower incidence of IAD (8.1%) compared to the group who received standard care (27.1%) (*p* = 0.003); however, our study shows better value of dry wipes. The observed efficacy of dry wipes, notwithstanding the low incidence of events recorded within this cohort, finds its physiological basis in the preservation of the acid mantle. By eliminating the need for rinsing and avoiding the use of traditional alkaline soaps, this method facilitates the maintenance of a pH level near 5.5, which is critical for the integrity of the lipid barrier and the enzymatic function of the stratum corneum. This pH stability mitigates bacterial overcolonization and protein degradation, processes that typically precede the onset of IAD. For institutions operating under stringent budgetary constraints, these findings demonstrate that the initial investment in specialized materials translates rapidly into a reduction in operational costs, stemming from a lower incidence of cutaneous complications and a decrease in nursing hours dedicated to complex wound care.

A doctoral thesis with some similarity to this one, but using a randomized controlled trial (RCT) [26] comparing a ZnO ointment and an NIBP with basic hygiene (soap and water), found no statistically significant differences between the intervention groups and the control group in the prevention of IAD. The specific results showed a relative risk of 0.54 for NIBP versus the control, with a *p* = 0.183 value, indicating that the difference was not statistically significant.

The quality and efficacy of absorbent products directly influence the prevention and treatment of IAD. Aspects such as design, absorbency, retention, and breathability are crucial [16,26,38]. Its use without clinical justification, without an adequate assessment of clinical needs, or in the absence of incontinence can cause unintentional harm to the patient, such as skin damage, risk of falls, or psychological dependence [39].

An improved diaper design, including breathable materials, can reduce skin occlusion and overhydration, thereby decreasing the risk of IAD [9,36,39,40]. Some diapers incorporate cellulose fibers that help reduce the pH on the surface of the skin or superabsorbent polymers that convert urine into gel, reducing exposure to air and helping to mitigate odor [9,39]. In addition, the size should be appropriate for the patient, as an ill-fitting absorbent around the legs and waist can increase the risk of IAD and leaks.

The composition of absorbents considered best for the prevention of IAD focuses on maintaining an acidic surface pH and reducing occlusion and excess moisture in the skin [41,42]. The spiral-curly fiber cellulose allows buffering of the pH of alkaline fluids (e.g., urine) and maintains a more acidic environment near the skin. This design, by interposing a special type of acidic cellulose, curly fiber or spiral, between the top layer (in contact with the skin) and the absorption core (containing superabsorbent polyacrylate) allows to achieve a surface pH of 4.6 that remained stable even after repeated wetting over a period of 5 h in contrast to the conventional composition that can reach values of 7.1 [41,43]. On the other hand, the introduction of superabsorbent polyacrylate polymers into the absorption core is essential. These compartmentalize liquids and prevent rewetting even under pressure, resulting in a drier skin microenvironment [41,42]. The addition of absorbers to permeable, breathable outer sheets reduces skin occlusion and is considered an advancement in the design of absorbents for incontinence control [41,42]. These compounds are present in the B brand of absorbents used in our study and obtained the best prevention results.

These same compounds could be present in brand A, although we are unable to confirm this because the data are sensitive and have not been provided by the manufacturers, and we can access only the product’s web information [44]. In addition, it is important to note that, as a limiting and influential factor, the degree of absorption of each scheduled absorbent: day, night, or supernight; since the number of changes in absorbents was taken into account, but not the degree of absorption that each one had.

In a 2018 RCT [26], the frequency of changes in absorbents was similar to that in our study: three daily absorbents, with a minimum of 2.84 and a maximum of 3.03. Also, finding a higher overall incidence of IAD, which we will discuss in the next section.

The frequency of absorbent product changes is a key factor in preventing IAD, although evidence on the optimal technique and frequency is limited [24]. Interestingly, some sources indicate that the risk of skin damage may increase with the frequency of soap and water cleaning, suggesting that routine washing after each episode of urinary incontinence may need to be re-evaluated and, when possible, reduced in frequency [9].

The effects of different change frequency regimes (less frequent, every eight hours, vs. frequent, every four hours) in institutionalized women [9,45] have been studied, with no significant differences in the incidence or severity of erythema/dermatitis between the groups.

Unexpectedly, no significant association was found between the frequency of absorbent changes and IAD incidence in the present study. A clinical explanation for this finding lies in the ‘paradox of hygiene’: frequent changes imply frequent skin manipulation. If changes are performed using abrasive hygiene care practices (such as friction with soap and water), the physical damage to the skin barrier may outweigh the benefits of removing moisture. Additionally, modern absorbents with superabsorbent polymers (SAPs) effectively isolate moisture from the skin, potentially making the absolute frequency of changes a less critical factor than the preventive capacity of the hygiene product used (e.g., 3-in-1 wipes).

As a final aspect to consider, current clinical practice in many long-term care facilities relies on traditional hygiene care practices (soap and water or soapy sponges) due to a lack of evidence-based protocols. Our findings suggest that this practice should be revised. The implementation of standardized skin care protocols is urgent, specifically recommending the replacement of soapy sponges with 3-in-1 containment wipes, which have shown to significantly delay the onset of IAD compared to traditional methods. These changes could reduce the incidence of dermatitis even in high-risk populations

Sociodemographic variables and their influence on the appearance of IAD.

Our study found no statistical significance (*p* = 0.81) in the association between the age of an incontinent patient and the occurrence of an IAD.

The prevalence and incidence of IAD tend to be higher in older people, and this is attributed to the fact that aging is a factor associated with incontinence and skin changes in older people, which make it more fragile and vulnerable, but it does not seem to be an isolated risk factor [9,25,26].

In relation to sex, the prevalence of urinary incontinence is higher in women, regardless of age, and due to anatomical differences, it leads to an increased frequency of IAD [9,24]. However, our study did not find a statistically significant relationship (*p* = 0.21).

Regarding the level of dependence, our study found no statistically significant association between dependence level and the development of an IAD. Nor has any direct or explicit relationship between the Barthel Index score and the appearance of IAD been found in the scientific literature. However, it has been identified that patients with higher degrees of functional and mental impairment have a higher probability of developing IAD [9,46]. This implies that greater dependence, as measured by scales such as the Barthel Index, could be indirectly associated.

The PAT is a scale designed to assess the risk of developing IAD. Our results showed a statistically significant association between the risk measured by the PAT scale and the incidence of IAD (*p* = 0.02). In fact, there are already preventive algorithms for IAD that use the PAT scale score to guide the frequency of diaper changes, for example, every two hours for high scores (8–12) and every four hours for medium scores (4–7) [47].

The original Braden scale was traditionally designed specifically to assess the risk of developing pressure injuries. However, a doctoral thesis was defended in 2016 [48] entitled “Predictive capacity of the risk assessment scales for pressure ulcers and other dependence-related injuries in the critically ill patient” and a subsequent article published in the Journal of Wound Care [49] focused on the predictive capacity of Braden and other scales (such as EMINA and EVARUCI) mainly for pressure injuries and dependence-related skin injuries in general. In the thesis, cutoff points were determined that offer the best balance between validity and predictive capacity for each scale. For example, the cutoff point that offered the best balance between validity and predictive capacity for the Braden scale in the context of Alba Roca Biosca’s doctoral thesis, as noted above, was 10.

Traditionally, moisture has been associated with the development of pressure injuries [36], and the Braden scale includes humidity as an extrinsic factor [50]. Factors in the Braden scale, such as constant or frequent skin moisture and friction and shear during repositioning or mobilization, increase the probability of IAD [9,46]. The Braden friction/shear subscale was associated with a 1.87-fold increase in the odds of IAD at admission per 1-point decrease in the score [51]. Altered sensory perception was also associated with IAD [51].

Therefore, a priori, the results of our research are unsurprising, with a statistically significant association (*p* = 0.004) between high Braden scale scores and the development of an IAD. The mean Braden score of patients who developed an IAD was 13.62.

## 6. Limitations

A limitation of this study is the reliance on univariate analyses. Multivariate adjustment was not feasible due to the small number of events in some hygiene care practice subgroups, which precluded robust modeling. Future studies with larger sample sizes are needed to confirm these independent associations.

Furthermore, advanced techniques to control for confounding, such as Propensity Score Matching or multivariate stratification, were considered but deemed unsuitable for this dataset. The limited sample size and the specific distribution of hygiene care practices across centers meant that such techniques would excessively reduce the effective sample size and statistical power. Therefore, the results are presented as observational associations that warrant confirmation in larger, multicenter trials.

Finally, multivariate adjustment (e.g., Cox regression) using risk scales like Braden or PAT was not performed. Due to the conceptual overlap and potential collinearity between these risk assessment tools, their simultaneous inclusion in a model with a limited number of events could yield unstable estimates, potentially masking the primary association with hygiene methods.

## 7. Conclusions

- The study found a statistically significant relationship between the Braden and PAT scales and the occurrence of IAD. This means that the greater the risk detected by these scales, the more lesions develop.
- No statistically significant relationship was found between age, sex, or Barthel Index and the development of IAD.
- The hygiene care practice significantly influenced the occurrence of IAD, showing that soapy wipes had the highest association with the occurrence of IAD, while dry wipes appear to be the most protective.
- In addition, the dry wipes hygiene method proved to be the most effective, as it presented the lowest cumulative risk and the longest time of onset of the event, in contrast to soap and water, where there was an earlier onset of IAD appearing throughout the follow-up.
- The brand of the absorbent was also significantly associated with the occurrence of IAD. The “B” brand prevented one IAD for every 6 patients compared to the “A” brand.
- The frequency of absorbent changes did not show a statistically significant association with the occurrence of IAD.

## Figures and Tables

**Table 1:** Frequency of qualitative sociodemographic variables (sex). Chi-square statistical test with Fisher’s correction.

IAD	Men	Women	*p*-Value
No IAD	30	84	0.21 (NS)
With IAD	17	33	

Source: Authors. (NS) = Not significant.

**Table 2:** Mean values of sociodemographic variables. Mann–Whitney U test.

	IAD (No N = 114) Yes (N = 50)	Average Value	Average Range	U de Mann–Whitney	*p*-Value
Age	No	85.65 (±) 9.5	83.09	2783.0	0.81 (NS)
	Yes	85.86 (±) 8.1	81.19		
Braden	No	15.08 (±) 2.5	89.47	2055.0	0.004 *
	Yes	13.62 (±) 2.6	66.60		
Barthel	No	26.10 (±) 24.8	85.01	2563.5	0.30 (NS)
	Yes	21.74 (±) 22.0	76.77		
PAT	No	4.93 (±) 1.0	77.23	2249.5	0.02 *
	Yes	5.34 (±) 1.2	94.51		

Source: Authors. (NS) = Not significant; (*) = Statistical significance.

**Table 3:** Summary of sociodemographic variables.

Variable Analyzed	Type of Association	Significance (*p*-Value)	Clinical Interpretation
Age	Not significant	*p* = 0.81	Older age, per se, is not a predictor; The functional state prevails.
Sex	Not significant	*p* = 0.58	Similar risk in men and women under hygiene protocols.
Braden scale	Significant	*p* = 0.004	Mean score of 13.62 in cases of IAD (Moderate Risk). There is an association between the detected risk and the appearance of an IAD.
PAT Scale	Significant	*p* = 0.02	Validates the intensity of contact with moisture as a risk.
Barthel Index	Not significant	*p* = 0.30	The degree of dependence or lack of autonomy is not statistically significant with the risk of IAD development.
Hygiene care practice	Very significant	*p* = 0.001	Dry wipes are the most protective method.
Absorbent Brand	Significant	*p* = 0.024	The quality of the material influences the appearance of IAD.
Absorbent Change Frequency	Not significant	*p* = 0.60	The cleaning technique and absorbent brand matter more than the frequency of change in isolation.

Source: Authors.

## Data Availability

The original contributions presented in this study are included in the article. Further inquiries can be directed to the corresponding author.
